# Exposure to MIV-150 from a High-Dose Intravaginal Ring Results in Limited Emergence of Drug Resistance Mutations in SHIV-RT Infected Rhesus Macaques

**DOI:** 10.1371/journal.pone.0089300

**Published:** 2014-02-27

**Authors:** Mayla Hsu, Brandon F. Keele, Meropi Aravantinou, Noa Krawczyk, Samantha Seidor, Ciby J. Abraham, Shimin Zhang, Aixa Rodriguez, Larisa Kizima, Nina Derby, Ninochka Jean-Pierre, Olga Mizenina, Agegnehu Gettie, Brooke Grasperge, James Blanchard, Michael J. Piatak, Jeffrey D. Lifson, José A. Fernández-Romero, Thomas M. Zydowsky, Melissa Robbiani

**Affiliations:** 1 Center for Biomedical Research, Population Council, New York, New York, United States of America; 2 AIDS and Cancer Virus Program, Leidos Biomedical Research, Inc. (formerly SAIC-Frederick, Inc.), Frederick National Laboratory, Frederick, Maryland, United States of America; 3 Aaron Diamond AIDS Research Center, Rockefeller University, New York, New York, United States of America; 4 Tulane National Primate Research Center, Covington, Louisiana, United States of America; University of Pittsburgh, United States of America

## Abstract

When microbicides used for HIV prevention contain antiretroviral drugs, there is concern for the potential emergence of drug-resistant HIV following use in infected individuals who are either unaware of their HIV infection status or who are aware but still choose to use the microbicide. Resistant virus could ultimately impact their responsiveness to treatment and/or result in subsequent transmission of drug-resistant virus. We tested whether drug resistance mutations (DRMs) would emerge in macaques infected with simian immunodeficiency virus expressing HIV reverse transcriptase (SHIV-RT) after sustained exposure to the potent non-nucleoside reverse transcriptase inhibitor (NNRTI) MIV-150 delivered via an intravaginal ring (IVR). We first treated 4 SHIV-RT-infected animals with daily intramuscular injections of MIV-150 over two 21 day (d) intervals separated by a 7 d drug hiatus. In all 4 animals, NNRTI DRMs (single and combinations) were detected within 14 d and expanded in proportion and diversity with time. Knowing that we could detect *in vivo* emergence of NNRTI DRMs in response to MIV-150, we then tested whether a high-dose MIV-150 IVR (loaded with >10 times the amount being used in a combination microbicide IVR in development) would select for resistance in 6 infected animals, modeling use of this prevention method by an HIV-infected woman. We previously demonstrated that this MIV-150 IVR provides significant protection against vaginal SHIV-RT challenge. Wearing the MIV-150 IVR for 56 d led to only 2 single DRMs in 2 of 6 animals (430 RT sequences analyzed total, 0.46%) from plasma and lymph nodes despite MIV-150 persisting in the plasma, vaginal fluids, and genital tissues. Only wild type virus sequences were detected in the genital tissues. These findings indicate a low probability for the emergence of DRMs after topical MIV-150 exposure and support the advancement of MIV-150-containing microbicides.

## Introduction

Development of viral resistance to antiretroviral (ARV) therapy for HIV has been a concern since even before the first anti-HIV drugs were approved. With good compliance to current first-line combination drug regimens, resistance is less common but remains a concern in settings where the drugs or drug combinations used and/or patient compliance are not optimal [1]–[3]. In addition to posing a significant clinical challenge, particularly in resource-limited settings [4], [5], transmission of drug-resistant virus could complicate control of the HIV pandemic [2], [6]–[9]. Prophylactic strategies such as Pre-Exposure Prophylaxis (PrEP) and microbicides, which are targeted toward HIV-uninfected individuals, could potentially be used by infected persons, some of whom are unaware of their HIV status [10], thereby enhancing the selection and transmission of drug-resistant HIV [11].

We have developed promising microbicide products containing the non-nucleoside reverse transcriptase inhibitor (NNRTI) MIV-150, an ARV that is not used therapeutically and is rapidly cleared [12]–[15]. Released from a carrageenan (CG) gel or an intravaginal ring (IVR), MIV-150 protects rhesus macaques from infection by a SHIV derived from SIVmac239 containing the reverse transcriptase of HIV-1_HXB2_ (SHIV-RT) [13], [16]. Protective efficacy is even higher when MIV-150 is coupled with zinc acetate (ZA) in our microbicide formulations [14], [15], [17] (Kizima et al., In revision). Moreover, the inclusion of non-ARV components with different mechanisms of action reduces the likelihood of resistance development. The MIV-150/ZA/CG (MZC) gel is advancing into clinical testing, and an MZC IVR, which would provide sustained delivery of MZC, is in preclinical development. In conjunction with providing female controlled HIV prevention options, IVRs may afford more consistent use and improved protection over methods requiring pericoital application [18], [19].

While MIV-150 resistant virus can be generated with difficulty using extended *in vitro* selection (Table 1 and Fernández-Romero, unpublished), MIV-150-containing microbicides have never selected for the transmission of drug-resistant SHIV-RTs in our *in vivo* macaque studies [12], [14], [15] (Kizima et al., In revision). The fact that the RT was sequenced during acute infection – when viral load was high and when the risk of resistance emerging might be increased – underscores the lack of resistance observed in these studies. However, examining the impact of topically delivered MIV-150 to already-infected animals has not been explored and poses a separate important question. The objective of this study was to determine whether use of MIV-150 IVRs in SHIV-RT+ animals would result in the emergence of viruses with DRMs, as a model of HIV+ humans who might be exposed to topical MIV-150. After demonstrating that systemically administered MIV-150 could drive the emergence of increasingly diverse single and combination DRMs in SHIV-RT+ animals over time, we utilized a high-dose MIV-150 IVR (containing >10 times the MIV-150 dose that is likely to be in the human-sized IVR) to assess the likelihood that a MIV-150-containing microbicide would fuel the emergence of DRMs *in vivo.* The MIV-150 IVR resulted in very low but measurable levels of MIV-150 in plasma and tissues, but even after 8 weeks (wks), rare single DRMs were only detected in the blood of one animal and the inguinal lymph node (LN) of another, but not in the cervical or vaginal mucosal tissues. This study provides the first data on the emergence of DRMs after topical ARV application in infected animals and underscores the potential for MIV-150-containing products to effectively limit HIV transmission without significant drug-resistance issues.

**Table 1:** Antiviral activity of MIV-150 against HIV isolates harboring distinct NNRTI-associated mutations.

HIV-1 isolate	NNRTI-associated mutations	Antiviral assay	IC_50_ nM (95% confidence interval)
NL4-3	WT	PBMC	0.6 (0.2 to 1.7)
29129-2	K103N	PBMC	0.5 (0.2 to 1)
MN	WT	TZM-bl	0.2 (0.18 to 0.25)
MN_NVP RES	Y181C	TZM-bl	0.1 (0.1 to 1.8)
MN_MIV-150 RES	L100I, K103N, Y181C	TZM-bl	> 100

## Materials and Methods

### MIV-150 and placebo formulations

MIV-150 was dissolved in N-methylpyrrolidinone (NMP) (Sigma Aldrich, St. Louis, MO) to a final concentration of 5 mg/ml and filtered through a 0.2 µM PTFE filter (Whatman, Piscataway, NJ) into sterile glass vials (Allergy Labs, Oklahoma City, OK). Daily intramuscular doses of MIV-150 were 0.5 mg/kg/day (0.1 ml/kg/day of the NMP solution), with placebo controls receiving 0.1 ml/kg/day of NMP.

### Formulation of IVRs

The 100 mg MIV-150 and placebo EVA rings were made as previously described [13].

### Virus stock

The SHIV-RT used for these studies was originally constructed by replacing the RT of SIVmac239 with that of HIV-1_HXB-2_ and characterized as described by Überla, et al [20]. Replication of this SHIV-RT was effectively inhibited by nevirapine [20] and MIV-150 [21]. SHIV-RT was provided for our use in microbicide studies evaluating MIV-150 by Disa Böttiger, Medivir AB. We have used stocks of this SHIV-RT for all our microbicide testing to date, growing and titering the virus in activated human peripheral blood mononuclear cells (PBMC) as previously described [12].

### Ethics statement

The Tulane National Primate Research Center (TNPRC) is an Association for Assessment and Accreditation of Laboratory Animal Care accredited facility (AAALAC #000594) in Covington, LA. Animal care is provided by a faculty of 8 veterinarians (7 ACLAM Diplomats) and 110 animal-care technicians, veterinary technicians, and enrichment staff. Post-IACUC approval monitoring by veterinary faculty and staff includes procedure evaluation, assessment of technical skill, and adherence to Standard Operative Procedures. All protocols were reviewed and approved by the Institutional Animal Care and Use Committee (IACUC) of TNPRC (OLAW animal welfare assurance number A4499-01, USDA registration number 72-R-0002) prior to initiation of the study, and veterinary faculty routinely monitored adherence to procedures and technical skill. All animal care procedures were compliant with the Animal Welfare Act and the Guide for the Care and Use of Laboratory Animals [22], [23]. Under these guidelines, animals were socially housed unless restricted by study design (i.e. after challenge until infection status was confirmed, and then animals were co-housed by infection status). Housing restrictions were scientifically justified and approved by the IACUC as part of protocol review. All animals in this study were fed commercially prepared monkey chow twice daily. Supplemental foods were provided in the form of fruit, vegetables, and foraging treats as part of the TNPRC environmental enrichment program. The TNPRC environmental enrichment program is reviewed and approved by the IACUC semiannually. Extensive efforts are made to find compatible pairs for every study group with additional environmental enrichment of housing space through a variety of food supplements and physical complexity of the environment. A team of 11 behavioral scientists monitors the well being of the animals and provides direct support to minimize stress during the study period. Veterinarians are available 24 h a day to provide emergency care. The TNPRC Division of Veterinary Medicine has established procedures to minimize pain and distress through several means. The use of preemptive and post procedural analgesia is required for procedures that would likely cause more than momentary pain or distress in humans undergoing the same procedure. Any deviation from the administration of analgesics according to this policy requires adequate scientific justification from the investigator and approval by the IACUC. The TNPRC has a written endpoint policy to minimize potential pain and distress experienced by animals, which addresses limits on weight loss, appetite, tumor size, response to medical intervention, activity, and a number of other clinical signs relevant to the animal species. If an animal becomes ill and/or meets the criteria for the IACUC approved endpoint policy, it is euthanized using methods consistent with recommendations of the American Veterinary Medical Association (AVMA) Guidelines for the Euthanasia of Animals, 2013.

### Animal treatments and challenge

Female adult Indian rhesus macaques (*Macaca mulatta*) infected with SHIV-RT (for 6–17 months; previously challenged intravaginally with 1000 TCID_50_ SHIV-RT [13]–[15], [24]) were housed at the TNPRC. Animals were selected for the study based on plasma viral loads to ensure that the animals had at least approximately 10^4^ RNA copies/ml at the initiation of the studies. Initially, animals received daily intramuscular injections for 7 days (d) with 0.5 mg/kg/d MIV-150 in NMP or NMP placebo, but no DRMs were detected in plasma virus RNA on d 8 (not shown). To increase the potential for selection of DRMs, 9 months later, the dosing was increased to daily injections of MIV-150 (0.5 mg/kg/d) or NMP placebo for 3 wks, followed by a 1-wk drug hiatus, and then another 3-wk period of daily MIV-150 or placebo injections. Blood (<10 ml/kg/month), LNs, vaginal swabs, and cervical and vaginal pinch biopsies were collected at various time-points and were shipped to the Center for Biomedical Research, Population Council, NY by overnight courier. Animals were anesthetized with tiletimine/zolazepam (8 mg/kg body weight) prior to blood draws and LN biopsy and treated with buprenorophine (0.01 mg/kg body weight) for analgesia. Animals that became ill or met the TNPRC IACUC-approved endpoint criteria for minimizing pain and distress were euthanized by sedation followed by barbiturate injection, which is the recommended method of euthanasia of rhesus macaques by the American Veterinary Medical Association (AVMA) Guidelines for the Euthanasia of Animals, 2013.

To determine whether MIV-150 IVRs selected for DRMs, a separate group of SHIV-RT-infected animals was selected. Animals were injected with 30 mg intramuscular Depo-Provera 5 wks prior to IVR insertion to mimic the conditions and timing of virus challenge in microbicide IVR efficacy studies [13], [24]. Six animals wore 100 mg MIV-150 IVRs, and 2 animals wore placebo IVRs for a total of 8 wks.

### Sample collection

Blood from challenged animals was collected in 7.5 ml EDTA vacutainer tubes, and plasma and peripheral blood mononuclear cells (PBMCs) were isolated [16], [25]. Axillary and inguinal LN mononuclear cells (LNMCs) were isolated by passing LN cell suspensions through 70 µm sieves and washing with cold PBS. Cervical and vaginal tissues were obtained as described [13], cut into 3 mm×3 mm×3 mm pieces and snap-frozen at –80°C [13]. Vaginal swabs were collected and shipped as previously described [13]. The fluids were clarified by centrifugation at 1700 rpm for 10 minutes (min) in a table-top centrifuge before being aliquotted and frozen at –80°C until MIV-150 determination.

### MIV-150 determination in plasma, swabs and tissues

Radioimmunoassay (RIA) was used to quantify MIV-150 in the swabs and plasma of animals in the intramuscular injection study and in the swabs of animals in the IVR study (LLOQ = 1 ng/ml or 2.7 nM) using an indirect extraction based assay adapted from Kumar, et al [15], [26]. We subsequently developed and validated a more sensitive liquid chromatography tandem mass spectrometry (LCMS/MS) method for MIV-150 determination in plasma (LLOQ = 20 pg/ml or 54 pM) and tissue (LLOQ = 111 fg from ∼20 mg of tissue) [14] (Kizima et al., In revision) that was used for the IVR study.

### MIV-150 susceptibility of *in vitro* selected NNRTI-resistant HIV

The anti-HIV activity of MIV-150 was measured against 5 different HIV isolates: HIV_NL4-3_ and HIV_29129-2_ [27] obtained through the NIH AIDS Reagent Program from Dr. Malcolm Martin (HIV_NL4-3_) and Dr. Robert W. Shafer (HIV_29129-2_), and HIV_MN_, HIV_MN_NVP RES_, and HIV_MN_MIV-150 RES_. Different concentrations of MIV-150 were added in triplicate to activated PBMCs (2×10^6^/ml) [28] or TZM-bl cells [29] 1 h before challenge with 100 TCID_50_ of HIV_NL4-3_ or HIV_29129-2_ (in PBMCs) or 200 infectious units of HIV_MN_, a nevirapine resistant HIV (HIV_MN_NVP RES_) or MIV-150 resistant HIV (HIV_MN_MIV-150 RES_, in TZM-bl cells). The 2 resistant viruses were selected from passages of HIV_MN_ in the presence of the indicated NNRTIs. In the PBMC assay, media was exchanged on d 1 and 4 post-infection, and supernatant p24 was measured on d 7 by ELISA (Zeptometrix, Buffalo, NY). The TZMbl assay was performed as previously described [29]. IC_50_ values were calculated using a dose-response-inhibition analysis on GraphPad Prism v5.0c software.

### Clonal sequencing

RNA was extracted from 1 ml plasma aliquots with QIAamp UltraSens Virus RNA kit (Qiagen, Valencia, CA) and reverse-transcribed using SuperScript III First-strand Supermix and random hexamer primer (Invitrogen, Carlsbad, CA). DNA was extracted from LN cell suspensions with QIAamp Blood and Tissue kit (Qiagen). PCR amplification was carried out to amplify the *pol* gene encoding the first ∼250 amino acids of RT with primers as previously described [15]. PCR products were purified with QIAquick gel extraction or QIAquick PCR purification kits (Qiagen). Clones were prepared by Zero-Blunt TOPO cloning (Invitrogen) and transformation of Top10 *E. coli* (Invitrogen). Individual bacterial colonies were grown in LB-broth (Invitrogen), and DNA was extracted with QIAprep Spin Miniprep kits (Qiagen). The presence of the RT gene was verified by restriction digestion and visualization on a 1% agarose gel, and positive clones were sent for sequencing (GeneWiz, South Plainfield, NJ) with primers specific to HIV_HxB2_ RT. Gene sequences were analyzed by Lasergene 10 (DNAStar, Madison, WI). Mutations were classified as NNRTI DRMs as defined by the Stanford University HIV Drug Resistance Database (http://hivdb.stanford.edu/) [30].

### Single genome amplification (SGA)

As a higher throughput method for determination of DRMs in SHIV-RT sequences, we used SGA to analyze virus from plasma, cells and tissues from the animals wearing IVRs. Virus RNA was extracted using QIAamp UltraSens Virus RNA kits (Qiagen) for plasma samples and phenol/chloroform extraction following tissue homogenization using Fast-Prep24 (MP Biomedicals) for tissue biopsy samples. RNA was immediately reverse-transcribed in 1× RT buffer consisting of 0.5 mM of each deoxynucleoside triphosphate, 5 mM dithiothreitol, 2 U/ml RNaseOUT (RNase inhibitor), 10 U/ml of SuperScript III RT, and 0.25 mM antisense primer SIV-Int-R1 5′- AAG CAA GGG AAA TAA GTG CTA TGC AGT AA-3′. The RT reaction was incubated at 50°C for 60 min, 55°C for 60 min and then heat-inactivated at 70°C for 15 min followed by treatment with 1 U of RNase H at 37°C for 20 min. *Pol* was amplified via limiting dilution PCR in which only one amplifiable molecule was present in each reaction as previously described for analysis of *Env* sequences [31]. PCR amplification was performed with 1× PCR buffer, 2 mM MgCl_2_, 0.2 mM of each deoxynucleoside triphosphate, 0.2 µM of each primer, and 0.025 U/µl Platinum Taq polymerase (Invitrogen) in a 20 µl reaction. First round PCR was performed with sense primer SIV-Pol-F1 5′-GGC AAT GCA GAG CCC CAA GAA GAC AGGG-3′ and antisense primer SIV-Int-R1 under the following conditions: 1 cycle of 94°C for 2 min, 35 cycles at 94°C for 15 sec, 55°C for 30 sec, and 72°C for 4 min, followed by a final extension of 72°C for 10 min. Next, 1 µl of the first-round PCR product was added to a second-round PCR reaction that included the sense primer SIV-Pol-F2 5′-GGG ATG CTG GAA ATG TGG AAA AAT GGA CC-3′ and antisense primer SIV-Int-R3 5′-CAC CTC TCT AGC CTC TCC GGT ATCC-3′ performed under the same conditions used for first-round PCR, but with a total of 45 cycles. Correct sized amplicons were identified by agarose gel electrophoresis and directly sequenced with second round PCR primers and 4 additional HIV-specific primers using BigDye Terminator technology (Invitrogen). To confirm PCR amplification from a single template, chromatograms were manually examined for multiple peaks, indicative of the presence of amplicons resulting from PCR-generated recombination events, or Taq polymerase errors. Mutations were classified as NNRTI DRMs as defined by the Stanford University HIV Drug Resistance Database (http://hivdb.stanford.edu/) [30]. The 643 RT sequences identified by SGA in this study were submitted to GenBank and assigned the numbers KF597883-KF598526.

### SHIV-RT plasma viral load

Plasma obtained from EDTA-treated whole blood was used as a source for determination of SIV *gag* RNA by quantitative RT-PCR assay [32]. The lower limit for detection was 30 RNA copies/ml plasma.

### Statistical analysis

Changes in mean viral load for animals treated with either placebo or MIV-150 were compared by paired t-test. Comparisons of area under the curve (AUC) for total viral load were by one-way ANOVA. P values less than 0.05 were considered statistically significant (GraphPad Prism 5.02, San Diego, CA).

## Results

### Systemic administration of MIV-150 to SHIV-RT-infected macaques drives the emergence of DRMs

MIV-150 is a potent NNRTI that we are developing as a combination microbicide containing also ZA and CG (MZC) [14], [15], [17] (Kizima, et al., In revision). As with other NNRTIs, HIV can develop resistance against MIV-150, but this is difficult to achieve, requiring 2–3 mutations in RT under extended *in vitro* selection (Table 1 and Fernández-Romero, et al, in preparation). Moreover, we have never detected the emergence of drug-resistant SHIV-RT in our *in vivo* macaque studies testing MIV-150-containing gels or IVRs [12]–[15] (Kizima et al., In revision). However, since it is possible that an HIV-infected woman may use an ARV-containing microbicide, it is important to evaluate the potential impact of a topically applied ARV in an infected individual. This can be readily explored utilizing SHIV-RT infected macaques.

**Figure 1:**
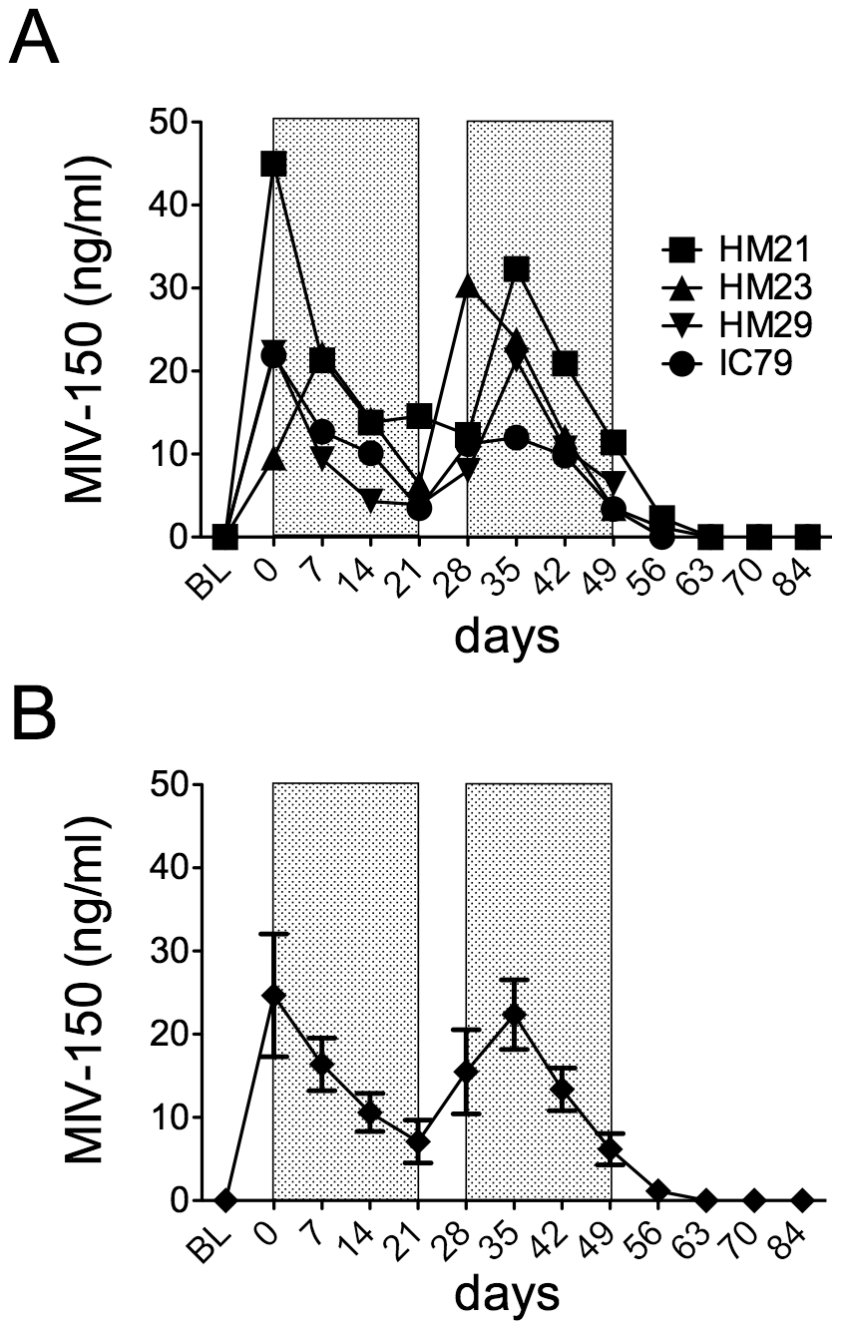
Plasma MIV-150 in animals receiving daily intramuscular injections. MIV-150 injections (0.5 mg/kg/d) were administered for two 21 d periods (with a 7 d break). MIV-150 levels were measured by RIA prior to (baseline, BL) dosing, as well as 3 h after the first injection of each 21 d regimen (d 0 and d 28) and then weekly thereafter just prior to each injection. The shaded areas indicate periods of daily MIV-150 injections. MIV-150 concentrations for individual animals are shown in A while mean concentrations (± SEM) are shown in B. MIV-150 was not detected in placebo-treated animals (not shown).

**Figure 2:**
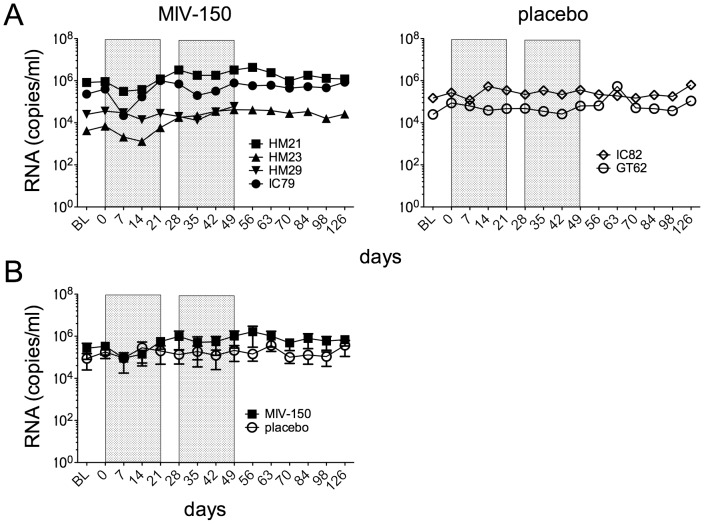
Plasma viral load in animals after systemic administration of MIV-150. Plasma virus levels were measured in the MIV-150 vs. placebo-treated animals over time. Viral load for individual animals is shown in A and mean viral loads (±SEM) are shown in B for the two treatment groups. The shaded areas indicate periods of daily injections.

Before delivering MIV-150 topically, we first verified that we could detect DRMs in infected animals exposed to systemic MIV-150. To maximize virus exposure to MIV-150, SHIV-RT+ animals were treated with intramuscular (i.m.) injections of MIV-150 (0.5 mg/kg/d), after which we sequenced plasma virus. We initially treated 4 animals for 7 d, but observed no DRMs in plasma virus on d 8 (not shown). We later re-treated the same animals under a dosing regimen of daily i.m. injections for two 21 d periods, separated by a 7 d treatment interruption. The strategy was deliberately non-therapeutic in order to enhance the emergence of mutations. Samples were taken 3 h after the first dose in each 21 d dosing period (d 0 and d 28) and then just prior to each subsequent injection. Plasma MIV-150 was detected immediately (3h post-injection shown as d 0 and d 28 in Figure 1) after injection and persisted over the next 21 d, exhibiting a similar pattern for all animals. Plasma MIV-150 did not accumulate over time or persist after the cessation of injections; instead plasma drug concentrations peaked 3 h after the first injection of each series and then rapidly declined towards baseline. Although MIV-150 levels were not measured just prior to starting the second set of injections on d 28, we expect that the levels were comparable to the similar time-point d 56, and thus low/undetectable since MIV-150 is typically rapidly cleared [12]–[15]. No plasma MIV-150 was detected in the 2 placebo-injected animals (not shown). A small decrease in the animals’ plasma viral loads during the first 7 to 14 d of MIV-150 treatment was not sustained (Figure 2). There was no significant difference between the mean viral load in MIV-150-treated animals at baseline (BL) vs. d 7 (p = 0.24), or in the plasma viral load AUC for animals treated with MIV-150 vs. placebo (p = 0.42).

Sequencing of plasma-derived viral RNA revealed that exclusively wild-type (wt) RT sequences were found in all 4 MIV-150-treated animals when sampled 7 d after beginning daily MIV-150 injections (Table 2). After 14 d, DRMs that reportedly confer diminished susceptibility to NNRTIs appeared although wt sequences were most prevalent in the 3 MIV-150-treated animals evaluated at this time-point. In the fourth animal, HM23, there was a limiting amount of virus (1.3×10^3^ RNA copies/ml) at d 14 (Figure 2A), and we were unable to amplify RNA for sequencing. The proportion of DRMs expanded during subsequent wks (most commonly at positions K101, K103, and Y181), and wt sequences declined to a minority species. Double and triple combinations of DRMs appeared as early as d 14 in plasma (HM21), and increased in proportion as well during the remaining wks of follow-up.

**Table 2:** Detection of DRMs after systemic treatment of infected macaques with MIV-150.

	Plasma genotype#						LN genotype#
MIV-150	day 7	day 14	day 21	day 49	day 56	day 63	day 49
HM21	wt (14/14)	wt (9/14)	wt (8/20)	wt (1/18)	wt (1/16)	I178L (1/16)	wt (5/20)
		K103R+I178L (1/14)	Y181C (7/20)	K101E (5/18)	K101E (4/16)	Y181C (11/16)	K101E (2/20)
		I178L (3/14)	I178L (4/20)	Y181C (10/18)	K101E+E138A+Y181C (1/16)	V179A+Y181C (1/16)	Y181C (12/20)
		Y181C (1/14)	E138K (1/20)	K101E+Y181C (1/18)	K101E+Y181C (1/16)	K101E+Y181C (1/16)	K101E+Y181C (1/20)
				I178L+Y181C (1/18)	Y181C (9/16)	K101E+I178L (1/16)	
						K103N (1/16)	
HM23	wt (20/20)	N/D*	K101E (16/22)	K101E (1/19)	K101E (15/18)	wt (1/15)	wt (4/19)
			E138K (5/22)	K103N (12/19)	K103N (3/18)	K103N (14/15)	K101E (6/19)
			I178L (1/22)	K103N+Y181C (1/19)			K103N (9/19)
				Y181C (3/19)			
				E138K (2/19)			
HM29	wt (19/19)	wt (8/14)	wt (16/20)	I178V+E138K (20/20)	sAIDS**	sAIDS**	wt (18/22)
		K103N (1/14)	I178V (3/20)				K103N (3/22)
		I178V (5/14)	E138K (1/20)				I178V (1/22)
IC79	wt (24/24)	wt (9/14)	wt (5/20)	K103N (14/20)	K103N (15/16)	K103N (8/16)	wt (5/15)
		Y181C (4/14)	K101E (1/20)	Y181C (2/20)	K103N+V179L (1/16)	K103N+Y181I (4/16)	K103N (7/15)
		Y188H (1/14)	K103N (11/20)	Y181I (2/20)		K103N+V179I (4/16)	I178V (1/15)
			Y181C (1/20)	K103N+I178L (1/20)			K103N+Y181C (1/15)
			E138K (2/20)	K103N+Y181C (1/20)			K101E+V108I+I178V (1/15)
**Placebo**							
GT62				wt (9/9)			wt (9/9)
IC82				wt (10/10)			wt (10/10)

# number of sequences positive for the indicated substitution over the number tested by clonal sequencing, *not done; virus copy number was too low; **animal euthanized with sAIDS at day 49; wt  =  wild type.

To determine whether DRMs emerged in LNs as well as in plasma, we sequenced virus DNA in LNMCs at d 49 (Table 2) after completion of both rounds of MIV-150 injections when detection of DRMs was most likely. A higher proportion of wt sequences were found in LNMCs than in plasma at the same time-point (Table 2). Two placebo-injected animals (GT62 and IC82) had exclusively wt sequences at d 49 in both plasma and LNMCs. Additional SGA of IC82 plasma virus RNA confirmed the sole presence of wt sequence at all time-points tested (d 7 14/14, d 14 12/12, d 21 6/6, d 42 16/16, and d 56 3/3) (data not shown). Thus, we did not carry out further sequence analysis from these animals.

### Rare DRMs detected in SHIV-RT+ animals wearing a high-dose MIV-150 IVR

Having demonstrated that we could detect NNRTI DRMs in response to sub-optimal *in vivo* MIV-150 treatment, we assessed whether sustained release of MIV-150 locally from the high-dose MIV-150 IVR would drive the emergence of DRMs. Depo-Provera-treated animals had 100 mg MIV-150 vs. placebo IVRs inserted for 56 d. Blood was collected over the first 42 d but was not available on d 56. One day after IVR removal, the animals were euthanized, and reproductive tract tissues and LNs were collected.

**Figure 3:**
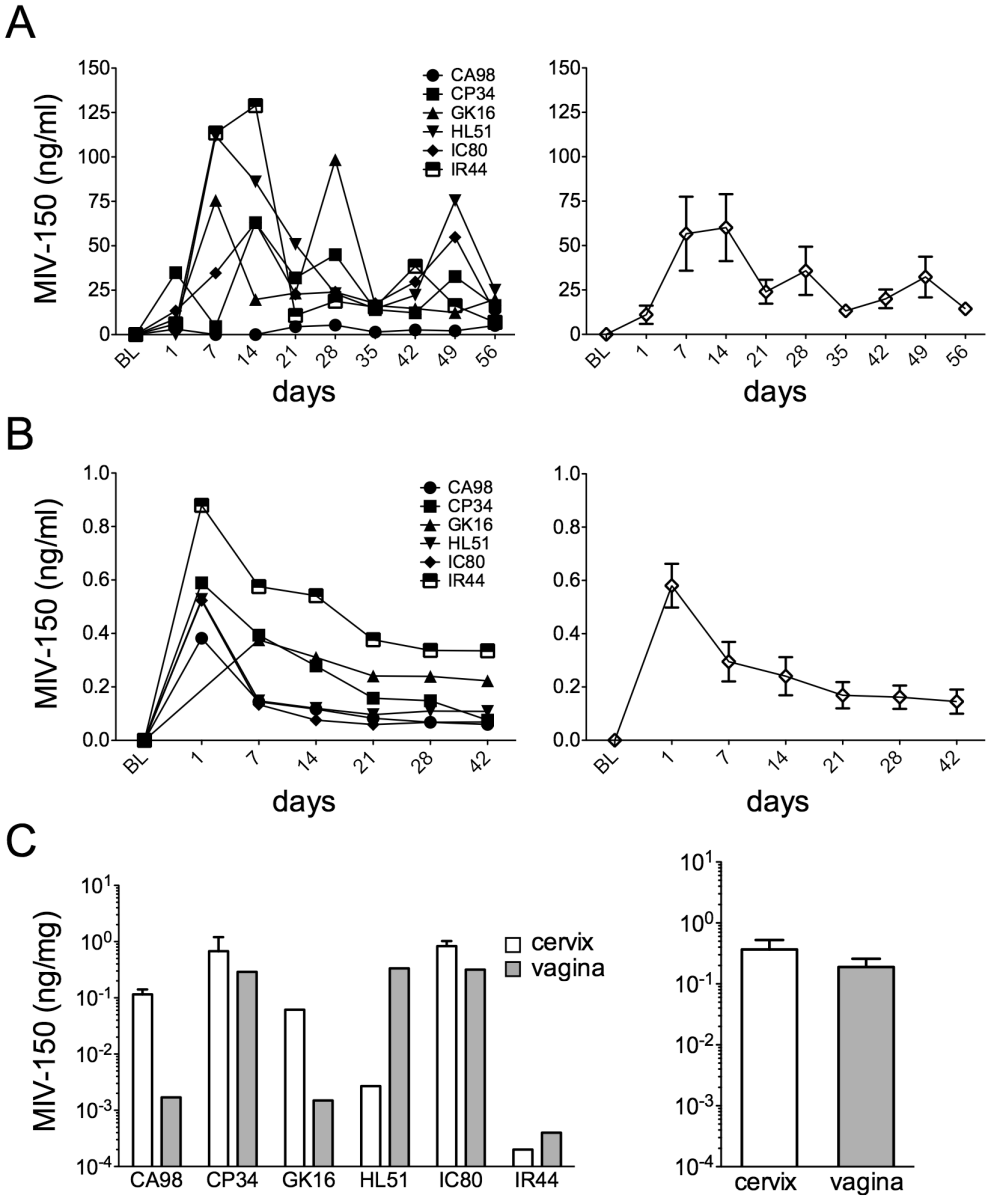
Sustained *in vivo* release of MIV-150 from IVRs for 56 days. Depo-Provera-treated SHIV-RT+ animals had 100 mg MIV-150 IVRs inserted for 56 d. Blood and vaginal swabs were collected prior to (BL) and after IVR insertion. Cervical and vaginal tissues were collected 1 d after IVR removal. MIV-150 levels in the vaginal swabs (A; measured by RIA) and plasma (B; measured by LCMS/MS) are shown for each animal (left panels) and the mean values for all animals (±SEM; right panels) over time. (C) MIV-150 levels in cervical and vaginal tissues (measured by LCMS/MS) are shown for each animal (left panel; 1-2 pieces of tissue measured per animal, mean±SEM shown for duplicates) and the means (±SEM, for all animals) for each tissue are shown in the right panel. Animals wearing placebo IVRs were negative for MIV-150 in swabs, plasma and tissues (not shown).

As expected from previous studies in which the MIV-150 IVRs were inserted 5 wks after Depo-Provera treatment [13], MIV-150 was detected in the swabs after 1 d and increased over the following 14 d (Figure 3A), declining by d 21 to a plateau that was sustained through the end of the study on d 56. These *in vivo* levels are similar to the 28 d *in vitro* release profile of MIV-150 from a 100 mg MIV-150 IVR using a non-sink release medium (i.e., MIV-150 release is limited by its solubility in the release medium) [13]. Using the LCMS/MS approach, which is more sensitive than the RIA used in previous studies (LLOQ of 20 pg/ml vs. 1 ng/ml [13]), we were able to measure MIV-150 in plasma. The highest levels of MIV-150 were detected after 1 d, lowering to plateau levels after 21 d (Figure 3B). Although variable between animals, MIV-150 was detected within the vaginal and cervical tissues 1 d after IVR removal (Figure 3C). In the 2 animals wearing placebo IVRs (DR51 and FL97), no MIV-150 was detected in vaginal swabs, plasma or tissues (not shown).

**Figure 4:**
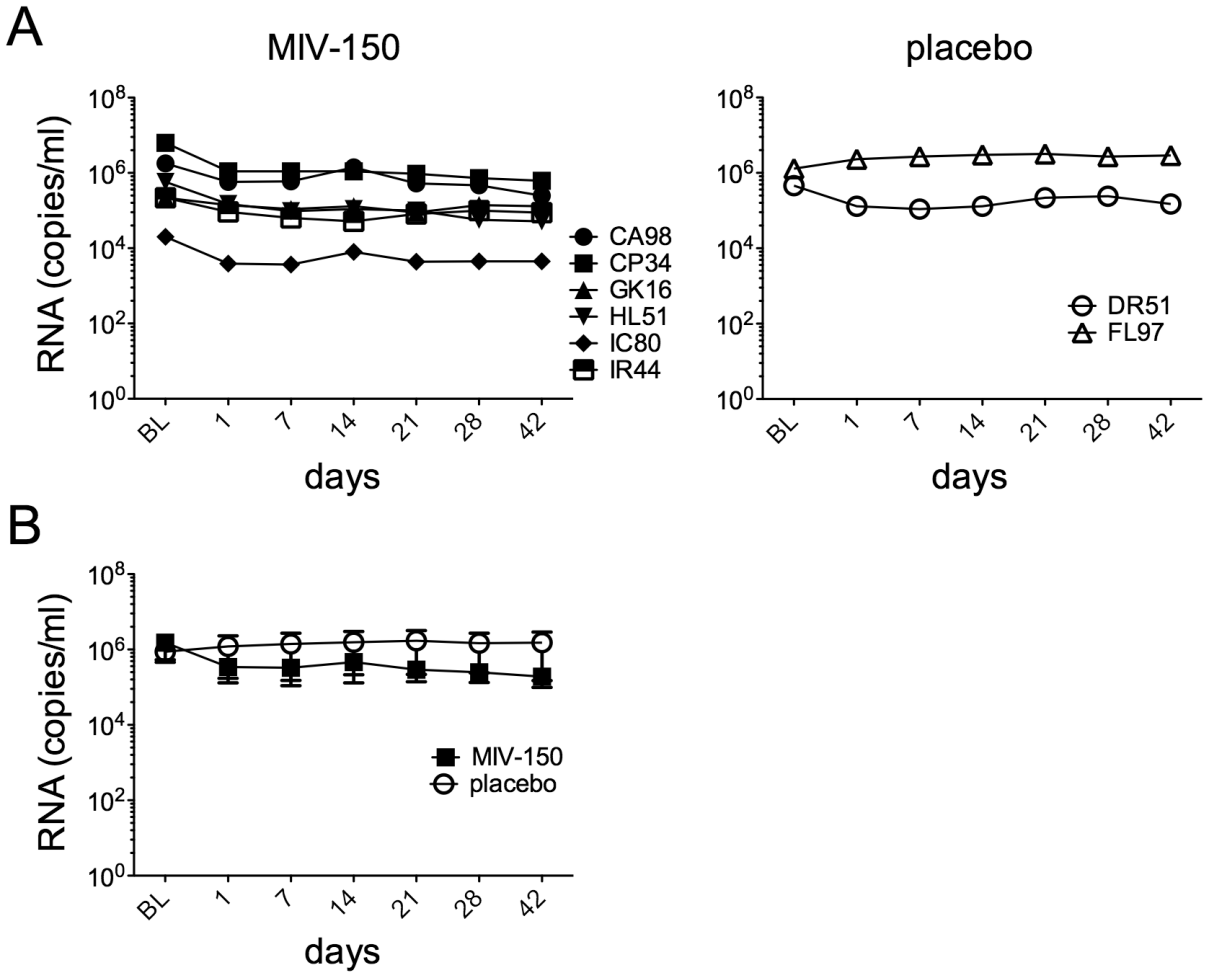
MIV-150 IVRs worn for 56 days have limited impact on plasma viremia. Plasma viral loads for individual animals are shown in A and mean viral loads (±SEM) are shown for both treatment groups in B.

Since plasma MIV-150 concentrations never exceeded 1 ng/ml while the animals wore MIV-150 IVRs, it is not surprising that the small decline in mean viral load between BL and 7 d post-insertion was insignificant (1.5×10^6^ to 3.3×10^5^ RNA copies/ml, p = 0.21), and there was no significant difference in AUC for mean viral load between placebo and MIV-150 IVR animals (Figure 4; p = 0.57).

To determine whether the sustained topical delivery of MIV-150 from IVRs drove the emergence of DRMs systemically and/or locally, we performed SGA on virus RNA obtained from plasma, PBMCs, LNMCs, and cervical and vaginal tissue (Table 3). In the final blood sample obtained (d 42), we found that all MIV-150-treated animals except IR44 had solely wt RT sequences in their plasma. In IR44, 1/27 plasma sequences had a Y181I substitution although the sequences from IR44’s PBMCs were exclusively wt (29/29). Further study of samples from this animal found only wt sequences in mesenteric, inguinal, and axial LNMCs at the d 57 time-point, indicating that the Y181I mutation was a rare species in this animal (1 mutant out of 145 genomes sequenced in blood and LNs post-ring-insertion). Because inguinal LNs drain the genital tract where the IVR was worn, we examined virus sequences from this tissue in all animals. Only CP34 had a single E138K mutation (1/30 sequences) in virus sequenced from the inguinal LN and had only wt sequences in mesenteric LN (1 mutant out of 63 genomes sequenced in blood and LNs post-ring-insertion). All other animals had completely wt LN sequences. Analysis of the cervical and vaginal tissue was more challenging due to difficulties in isolating viral RNA from these tissues. However, in the samples from which virus could be sequenced, only wt sequences were detected, including those from IR44 and CP34. There were too few animals with mutations to perform a correlation analysis, but notably, MIV-150 level in the blood was highest overall in IR44 while CP34’s mucosal tissue MIV-150 level was one of the highest (Figure 3).

**Table 3:** MIV-150 IVRs do not drive rapid emergence of DRMs in infected macaques.

	Plasma genotype#		PBMC genotype#	Day 57 LNMC genotype#			Day 57 tissue genotype#	
	Baseline	day 42	day 42	inguinal	mesenteric	axial	cervix	vagina
**MIV-150**								
CA98	wt (14/14)	wt (25/25)	ND	wt (29/29)	ND	ND	NA	wt (1/1)
CP34	wt (9/9)	wt (21/21)	ND	E138K (1/30), wt (29/30)	wt (12/12)	ND	wt (11/11)	wt (11/11)
GK16	wt (14/14)	wt (12/12)	ND	wt (11/11)	ND	ND	NA	NA
HL51	wt (15/15)	wt (23/23)	ND	wt (30/30)	ND	ND	wt (4/4)	wt (1/1)
IC80	wt (10/10)	wt (29/29)	ND	wt (28/28)	ND	ND	NA	NA
IR44	wt (16/16)	Y181I (1/27) wt (26/27)	wt (29/29)	wt (29/29)	wt (30/30)	wt (30/30)	NA	wt (7/7)
**Placebo**								
DR51	wt (10/10)	wt (28/28)	ND	wt (30/30)	ND	ND	NA	wt (3/3)
FL97	wt (14/14)	wt (7/7)	ND	wt (9/9)	ND	ND	NA	NA

#number of sequences positive for the indicated substitution over the number tested by SGA; ND = not done; NA = not amplifiable; wt  =  wild-type.

Taken together, these data suggest that the low levels of systemic MIV-150 reached after use of a high-dose MIV-150 IVR (carrying >10-times the MIV-150 planned for the human-sized MZC IVR) in infected macaques for 56 d result in few DRMs occurring at very low frequencies and in few compartments.

## Discussion

The MZC combination microbicide has been shown to be highly effective against vaginal and rectal SHIV-RT infection of macaques [14], [15], [17] (Kizima et al, In revision) and HSV-2 and HPV vaginal and rectal infections of mice (Kizima et al, In revision). This positions MZC as a promising combination microbicide to test in humans, and the MZC gel is advancing to clinical testing in 2014. We are also developing a MZC IVR to deliver this potent and broad-acting antiviral combination via a sustained delivery method. Macaques that have become infected in the presence of MIV-150-containing microbicide gels or IVRs harbored wt SHIV-RT at peak viremia [12]–[15] (Kizima et al, In revision), even when MIV-150 was present during acute infection in the MIV-150 IVR study [13]. Similar observations have been made with other ARV-containing formulations [33], [34]. Although microbicides are designed to prevent HIV infection, they might be used by HIV-infected individuals who may not know their HIV status [10] or who are HIV-infected and still choose to use the product, thereby possibly increasing the emergence and transmission of drug-resistant HIV [11]. To determine whether long-term topical application of MIV-150 could drive the emergence of DRMs in infected individuals, we inserted a prototype high-dose MIV-150 IVR into SHIV-RT+ macaques for 56 d and sequenced the virus isolated from the blood and tissues. Proof-of-concept studies had previously demonstrated that this high-dose MIV-150 IVR, which significantly protected Depo-Provera-treated macaques against vaginal SHIV-RT infection [13], loaded cervical and vaginal tissues with similar amounts of MIV-150 as observed in this study. Subsequent use of LCMS (LLOQ 20 pg/ml) to measure plasma MIV-150 levels in the animals from the efficacy study [13] revealed comparable levels and kinetics to those reported herein for the same time period (Robbiani and Zydowsky, unpublished).

In order to first demonstrate that we could detect DRMs *in vivo*, we administered MIV-150 in a non-therapeutic, systemic i.m. dosing regimen to enhance the emergence of NNRTI-driven mutations. Daily dosing resulted in a high but transient level of MIV-150 in the plasma after i.m. injection. The observed decline of MIV-150 during continued treatment in rhesus monkeys likely reflects the induction of drug-metabolizing enzymes by MIV-150. Human CYP3A4 has been implicated in metabolism of MIV-150 (personal communication Disa Böttiger and Bo Öberg) and other NNRTIs, and was recently shown to be induced by rilpivirine[35]. Coincident with a high level of MIV-150 in plasma after i.m. injections, we observed a large number of NNRTI-associated DRMs in the plasma SHIV-RT sequences, the most common of which (K103N and Y181C) are associated with high-level resistance to first-generation NNRTIs such as nevirapine and efavirenz, and K101E, which is associated with high level resistance to nevirapine and the latest generation NNRTIs (etravirine and rilpivirine) [30] (http://hivdb.stanford.edu/). Although we have shown that MIV-150 remains fully active against viruses containing these single DRMs through its advanced mode of binding (filling the NNRTI binding pocket distinctly from early generation NNRTIs) (Table 1 and Fernández-Romero, et al., in preparation), MIV-150 does apply pressure to these sites, pressuring the virus to mutate at these positions. Thus, it is not surprising that single DRMs arose and were sustained during the course of the i.m. study. The emergence of single DRMs followed by double and triple combinations highlights the pathway by which the virus evolves to evade drug pressure.

Of note in this first study was animal HM29. Despite having one of the lowest plasma MIV-150 concentrations, HM29 developed a unique DRM profile with I178V appearing in 5/14 clones within 2 wks of initiating MIV-150 therapy and the double mutation I178V+E138K coming to dominate 20/20 RT clones sequenced at 7 wks. To our knowledge, I178 has not been described in the literature as an important location for NNRTI-associated mutations (although all i.m. MIV-150 treated animals had at least a single clone containing a mutation at this position over the course of the study). I178 neighbors the common moderate resistance inducing V179D/E/F. It also neighbors Y181C, which has been shown to be incompatible with E138K *in vitro* [36] and was uniquely absent in the plasma of HM29. HM29 was also the only animal in the study to succumb to sAIDS (coincident with dominance of I178V+E138K at wk 7) though this animal’s peripheral CD4 count was not abnormally low (215 one month before euthanasia and 197 on the date of euthanasia) and plasma viral load was not high. HM29 did, however, exhibit consistent symptoms including diarrhea, weight loss, and wasting. Whether or not the establishment of this unique dominant genotype was linked to the development of sAIDS remains unknown.

Although viruses were not isolated from macaque plasma for phenotypic characterization, our *in vitro* data show that isolates harboring individual mutations (Y181C and K103N) are still susceptible to MIV-150, and only mutants containing 2 or more NNRTI-related mutations (obtained weeks after selection) are resistant to MIV-150 (Table 1 and Fernández-Romero, et al., in preparation). An interesting finding was the discrepancy between the DRMs observed after MIV-150 selection *in vitro* and in the i.m. MIV-150 study *in vivo*. *In vitro* selection led to the dominant emergence of the triple combination Y181C, K103N, and L100I while *in vivo*, we observed dominance of the individual mutations Y181C, K103N, and K101E and complete absence of L100I. The reason for these differences is unclear but may relate to characteristics of the parent viruses (HIV-1_MN_ vs. SHIV-RT) as well as to other differences between the *in vitro* and *in vivo* scenarios, such as viral fitness costs. Moreover, our i.m. *in vivo* data demonstrate that there are several pathways the virus takes in an effort to achieve a resistant phenotype. Published work also shows the unique pathways to resistance to other RT inhibitors [37], [38] as well as the incompatibility of certain DRM combinations, which might influence the eventual resistant genotype [36]. As previously observed after ARV treatment of SHIV-infected macaques, the proportion of plasma sequences with DRMs increased with time on drug [39] and with unsuppressed viral replication [40]. We found a higher percentage of wt sequences in LN DNA than in plasma, suggestive of archived integrated sequences that were not subject to the selective pressure of actively replicating virus; however because PBMC virus was not analyzed, we cannot compare cell-associated virus from these two compartments. Moreover, MIV-150 was not quantified in the LNs to address whether the persistence of more wt virus also correlated with lower drug levels.

Having shown that significant but sub-therapeutic levels of systemic MIV-150 could drive the emergence of DRMs, we next studied the impact of topically delivered MIV-150 by inserting the high-dose MIV-150 IVR into SHIV-RT+ macaques for 56 d. Although blood levels were considerably lower than those after i.m. MIV-150 dosing (<1 ng/ml at peak post IVR vs. ∼20–25 ng/ml at peak post i.m. injection), we could detect MIV-150 in plasma, vaginal swabs, and cervical and vaginal tissues. DRMs were observed in a minority of sequences in only 2/6 animals; 1 out of a total of 85 sequences for CP34 and 1 out of a total of 152 sequences for IR44 post-IVR insertion. These 2 animals had the highest and second-highest plasma MIV-150, respectively, while no DRMs (in a total of 193 sequences) were observed in the other 4 animals with lower MIV-150 levels. The quantity of systemic NNRTI may therefore correlate with the likelihood of DRM emergence. Indeed, animals exposed to i.m. MIV-150, in which several DRMs were observed, had mean peak plasma MIV-150 concentrations approximately 40 times higher than in animals wearing MIV-150 IVRs.

Only single mutations (E138K and Y181I) were detected in macaques wearing the high-dose MIV-150 IVR during the time period examined. The E138K mutation is associated with intermediate resistance to rilpivirine and potential low-level resistance to efavirenz, etravirine and nevirapine, and in combination with other DRMs, may increase resistance to other NNRTIs [41], [42] (http://hivdb.stanford.edu/). This mutation emerged in animals treated i.m. with MIV-150 and came to dominate in combination with I178V in one animal (HM29), indicating that E138K is likely on the pathway of viral evolution to evade MIV-150 pressure. Recent data show that despite inducing lower level resistance than other mutations at this position (A, G, Q), E138K is selected rapidly in response to drug pressure and preferentially, reflecting the G to A mutation bias of the HIV RT and possibly explaining why this DRM arose instead of other more potent ones in IVR-wearing animals [43]. *In vitro,* we have observed E138G in combination with other DRMs, but never singly in response to MIV-150. The Y181I mutation, which may confer high-level etravirine, rilpivirine, and nevirapine resistance, and intermediate resistance to efavirenz (http://hivdb.stanford.edu/), has not been observed singly in our *in vitro* studies (Fernández-Romero et al., in preparation) although it did appear as a minority species after i.m. MIV-150 injection. However, the low frequency of this mutation, which was observed in only 1/430 sequences (0.23%) from 1/6 animals treated with a MIV-150 IVR, suggests that in a parallel HIV-infected human scenario, its occurrence would be quite limited. In fact, the E138K and Y181I mutations occur in ≤1% NNRTI-resistant samples from HIV infected individuals [44], and studies on the *in vitro* selection of HIV resistance to MIV-150 revealed that at least 2 mutations are required for MIV-150 resistance (Table 1 and Fernández-Romero et al., in preparation). Importantly, we cannot rule out that more DRMs (alone and possibly in combination) might have been detected if the animals were exposed to MIV-150 for a longer time, if the IVRs were used intermittently, or if more sensitive and extensive sampling (e.g., quantitative allele specific PCR and/or deep sequencing) methods were used. However, the finding that such a limited repertoire of DRMs arose in animals wearing high-dose monotherapy IVRs suggests that use of combination products with lower ARV levels, such as the MZC combination IVR under development by our group, will invite even less resistance. Similar studies to those described herein will need to be carried out with our final formulations to draw definitive conclusions.

A limitation of this study is that clonal-based sequencing may not be sensitive enough to detect very rare DRMs that may be present before exposure to MIV-150, and in fact, made it impossible to determine whether the DRMs originated from pre-existing minority variants or were a result of error-prone virus replication [45], [46]. Additionally, in the absence of virus phenotyping, we are unable to determine whether the two DRMs that occurred in such low frequency in IVR-wearing animals were, in fact, from infectious viruses. If so, their replicative fitness compared to wt virus would be interesting to determine since some HIVs with DRMs may have attenuated growth [47], [48] and then acquire compensatory mutations that restore fitness [49], [50].

The principal aim of the study was to determine whether sustained exposure to topically applied MIV-150 would drive the emergence of resistant variants. Our results indicate that the probability of that happening is very low. The MIV-150 IVR tested contains approximately 10-fold more MIV-150 than we expect to load in a human sized MZC IVR, which further minimizes the potential for DRM emergence. Using a nonhuman primate model of HIV infection, we have shown that the MIV-150 IVR is acceptable in terms of the emergence of drug resistance, thus advancing MIV-150-containing IVRs (e.g. MZC IVR) as a microbicide strategy.
